# RESILIENCE COMPONENTS, POSTOPERATIVE COMPLICATIONS, AND QUALITY OF LIFE DETERIORATION AFTER PANCREATECTOMY

**DOI:** 10.1093/geroni/igae098.4067

**Published:** 2024-12-31

**Authors:** Jung-Yeon Choi, Kwang-il Kim

**Affiliations:** Seoul National University Bundang Hospital, Seongnam, Kyonggi-do, Republic of Korea; Seoul National University College of Medicine, Seoul National University, Seoul, Republic of Korea

## Abstract

**Background:**

Resilience, the ability to adapt positively to adversity, remains a multisystemic process with no standardized objective measurement methods. The aim of this study was to identify the association between resilience components and frailty, postoperative complications, and quality of life changes after pancreatectomy in older patients.

**Methods:**

This study evaluated older patients (aged ≥ 65) scheduled for pancreatectomy between August 2020 and December 2023. Patients who underwent a Comprehensive Geriatric Assessment and signed informed consent were included. Frailty was determined by multidimensional frailty score more than 5. Neurohumoral resilience was measured using the ACTH stimulation test, cardiovascular autonomic function using orthostatic blood pressure measurement, and cognitive-motor function using dual-task gait tests. The primary outcome was postoperative complications, and the secondary outcome was the deterioration in quality of life one year after pancreatectomy.

**Results:**

A total of 57 patients were included in the analysis. Among them, 17 (29.8%) were classified as frail, 10 patients (17.5%) experienced postoperative complications, and 12 patients (24.5%) had worsened quality of life after one year. Low blood pressure and slow usual gait speed was associated with frailty. Diminished cortisol responsiveness correlated with frailty and postoperative complications. Quality of life deterioration was associated with differences between dual-task (serial 7) gait speed and fast gait speed.

**Conclusion:**

This study highlights the potential association between multidomain resilience components, frailty, and clinical outcomes in older patients undergoing pancreatectomy. Future research should focus on developing robust, objective, and reliable resilience metrics for clinical use.

